# Machine learning based regional epidemic transmission risks precaution in digital society

**DOI:** 10.1038/s41598-022-24670-z

**Published:** 2022-11-28

**Authors:** Zhengyu Shi, Haoqi Qian, Yao Li, Fan Wu, Libo Wu

**Affiliations:** 1 School of Data Science, Fudan University, Shanghai, 200433 China; 2 Institute for Global Public Policy, Fudan University, Shanghai, 200433 China; 3 LSE-Fudan Research Centre for Global Public Policy, Fudan University, Shanghai, 200433 China; 4 MOE Laboratory for National Development and Intelligent Governance, Fudan University, Shanghai, 200433 China; 5 Shanghai Ideal Information Industry (Group) Co., Ltd, Fudan University, Shanghai, 200120 China; 6 Shanghai Public Health Clinical Center, Fudan University, Shanghai, 200032 China; 7 Key Laboratory of Medical Molecular Virology, Fudan University, Shanghai, 200032 China; 8 School of Economics, Fudan University, Shanghai, 200433 China; 9 Institute for Big Data, Fudan University, Shanghai, 200433 China

**Keywords:** Disease prevention, Health care economics, Health policy, Public health

## Abstract

The contact and interaction of human is considered to be one of the important factors affecting the epidemic transmission, and it is critical to model the heterogeneity of individual activities in epidemiological risk assessment. In digital society, massive data makes it possible to implement this idea on large scale. Here, we use the mobile phone signaling to track the users’ trajectories and construct contact network to describe the topology of daily contact between individuals dynamically. We show the spatiotemporal contact features of about 7.5 million mobile phone users during the outbreak of COVID-19 in Shanghai, China. Furthermore, the individual feature matrix extracted from contact network enables us to carry out the extreme event learning and predict the regional transmission risk, which can be further decomposed into the risk due to the inflow of people from epidemic hot zones and the risk due to people close contacts within the observing area. This method is much more flexible and adaptive, and can be taken as one of the epidemic precautions before the large-scale outbreak with high efficiency and low cost.

## Introduction

The international society was caught off guard by the unexpected outbreak of COVID-19 since the beginning of 2020^1^. Quite a few regions or even the whole country had adopted the lockdown policies in order to make the pandemic under control, and these non-pharmacological interventions had been demonstrated to be effective^2^. Meanwhile, these strict interventions had caused severe economic and social welfare losses as well. The world GDP growth rate has declined by 3.4% in 2020^3^ and around 81 percent of the global workforce was affected due to government responses to the pandemic^4^. However, when there lacks enough knowledge and information about the accurate transmission risks of COVID-19, the best policy response that policy makers may make is to try to cut off all possible transmission paths immediately.

Similar to other infectious diseases, spread of the COVID-19 is mainly resulted from direct, indirect and close contacts between people at the micro-level^5–7^, as well as from regional population flows at the macro-level^8–10^. Existing literature has shown that transmission risks are predictable for infectious diseases such as Severe Acute Respiratory Syndrome (SARS)^11^, Middle East Respiratory Syndrome (MERS)^12^, Ebola^13^ and flu^14^ by using various behavioral data such as search engine^15,16^, social media^17^ and wearable devices^18,19^. Assumptions behind these predictions focus more on the macro-level so that people’s contact behaviors are simplified as homogeneous parameters in traditional epidemiological models such as SI, SIR, SEIR and etc^14,20–23^. In such models, it is necessary to estimate the classic reproduction number accurately, that is, the number of secondary cases of infected individuals in the susceptible group^24–26^. But it is difficult to get the reproduction number which can fit the epidemic transmission evolution perfectly in the real world. The deviation between theory and reality can be explained by the differences of individual behaviors^27,28^. The neglect of the micro heterogeneity may lead to misestimating the regional epidemic risks. Moreover, the greater the difference of individual characteristics within the group, the greater the estimated error^29^.

Most of the early studies are limited to the small-scale with few personnel and low population flow such as families^30^, flights^31^ and hospitals^32^, but the premise assumption of this setting is too peculiar, so it is difficult to extend to the city and even the national level. The development of 5G and Internet of Things technology guarantees collection of individual trajectory data^33,34^. Therefore, many scholars try to obtain large-scale information about people contacts through wearable devices or mobile phones^35,36^, which creates more opportunities for further researches on epidemic transmission path and risk, seasonal fluctuation and spatial evolution and so on^37–39^. However, those previous epidemic studies focused more on the observed population migration between cities, base stations or some grid units, as well as the population density within a certain region^10,40^. This will inevitably lead to the fact that people in the same space are supposed to be homogeneous and static when considering regional risks, while ignoring the actual situation of dynamic contact among them.

In order to provide enough evidence at high-resolution level for policy makers to take targeted measures, heterogeneous individual level contact behaviors have been put more emphases on^41^, and some intelligent technology like big-data analytics^42^, artificial intelligence^43^, cloud computing^44^ and machine learning^45^ may provide better solutions. During the spreading period of COVID-19, many countries have tried to launch individual tracing systems and monitor potential transmission risks through smart phones^46,47^, and Apple and Google also developed COVID-19 Alert App jointly^48^. All of those applications need users be willing to install or use, otherwise they cannot offer the pandemic information for users. Since data coverage is more crucial for epidemic prevention, this invasive data acquisition way will reduce the prevention efficiency. Except for the individual trajectory, the contact topology is also quite important. As a straightforward scientific tool, complex network can effectively describe the dynamic contact topology between different individuals^49,50^, so as to obtain more micro scale discovery of epidemic transmission. For example, individual level contact network tends to show small-world and nonrandom graph properties^51,52^. These features reflect the fact that more complicated models^53,54^ are anticipated to investigate the micro mechanisms of infectious disease transmission. Then regional transmission risks can be more precisely identified by adopting comprehensive population flow pattern data. This type of bottom-up transmission risk modelling techniques has shown increasing importance in the policy making procedure in the public health field^55^. Another advantage of using big data in practice is that it can reduce unnecessary intrinsic risks in the traditional epidemiological surveys^56,57^. These risks are commonly caused by missing some part of objective information due to memory biases or dishonesties.

To overcome the aforementioned issues, we construct a novel contact network structure based on mobile phone signaling. It establishes a weighted contact topology network in a non-intrusive way and can reflect the difference of social interaction better. Since the data in the real world often suffer from the highly imbalanced distribution, the traditional methods can hardly deal with that^58–60^. For this reason, if conventional neural network is used to identify rare disease patients or high-risk virus carriers from a large number of negative people, the results will be seriously biased. Thus, after reconstructing the individual-centered contact feature, the neural network prediction of extreme events is carried out for each individual. In this study, we estimate the town-level transmission risks for COVID-19 in Shanghai based on a high-resolution contact network compiled from nearly 7.5 million mobile phone users. Individual level contact behaviors are modelled by using the machine learning method. Results show that this machine learning based bottom-up technique has great potential for identifying regional transmission risks. The interesting conclusions provide policy implications that unnecessary economic and welfare losses can be avoided by controlling the spread of infectious diseases in advance.

## Methods and data

### Contact strength

There are 94,733 Telecom base stations in Shanghai with an average coverage of 0.0669 square kilometers. We define that if two mobile phone signals interact with one base station at the same time slice τ, then the two individuals' trajectories have a coincidence. In this paper, the time slice τ is set to 1/12 hour. If individuals coincide with high-risk group, the risk of infection will increase, and consequently such contacts are called effective contacts; while the mutual contacts within the general group do not generate new risks of infection, such contacts are invalid. In order to simplify the contact analysis, it is necessary to concentrate on the effective contacts when identifying regional transmission risks of infectious diseases.

Furthermore, we constructed the contact strength to quantify the influence of effective contacts. Effective contact frequency is one of the determinants to increase the infectious transmission risks. The longer an individual has been exposed to the high-risk group, the more likely to be infected. Nevertheless, only considering the duration of effective contact is not enough. Since the individuals in high-risk group have been to different epidemic hot zones, the possibilities of carrying virus are distinct and we use a dynamic virus carrying risk coefficient to distinguish one from another. Thus, the contact strength can be calculated by the product of virus carrying risk coefficient from high-risk individual $h$ and effective contact frequency,

1 $$\omega_{h \to i,d} = t_{h,i,d} \times \gamma_{h,d}$$

where, $\omega_{h \to i,d}$ represents the contact strength between individual $i$ and individual $h$ on day $d$, which will be the weight of corresponding edge in the $d$ th contact network. $t_{h,i,d}$ is the times of effective contacts between individual $i$ and $h$ on the $d$ th day.

The virus carrying risk coefficient $\gamma_{h}$ of individual $h$ is determined by the epidemic hot zone with the highest risk coefficient in the recent viral incubation period $T_{virus}$. First of all, we define the epidemic infection density $\rho_{c}$ of epidemic hot city $c$ as the proportion of the cumulative number of confirmed cases in the permanent population,

2 $$\rho_{c} = \frac{{Nc_{c} }}{{Np_{c} }}$$

where, $Nc_{c}$ is the cumulative number of confirmed cases in city $c$, and $Np_{c}$ is the permanent resident population of city $c$ (unit: 10,000 people). We set infection density $\rho_{o}$ of the city with the transmission risk to be estimated as the baseline and adjust the other cities’ infection densities, so as to obtain the risk coefficient for travelling or living in city $c$,

3 $$r_{c} = \frac{{\rho_{c} }}{{\rho_{o} }}$$

where, $r_{c}$ is the risk coefficient of travelling or living in city $c$, and $r_{o}$ is the risk coefficient of the city to be estimated. Obviously, $r_{o} = 1$. Therefore, the $\gamma_{h,d}$ is equal to the maximum value of $r_{c}$ in the historical trajectory of individual $h$ counting down $T_{virus}$ from day $d$.

### Contact networks

**Figure 1:**
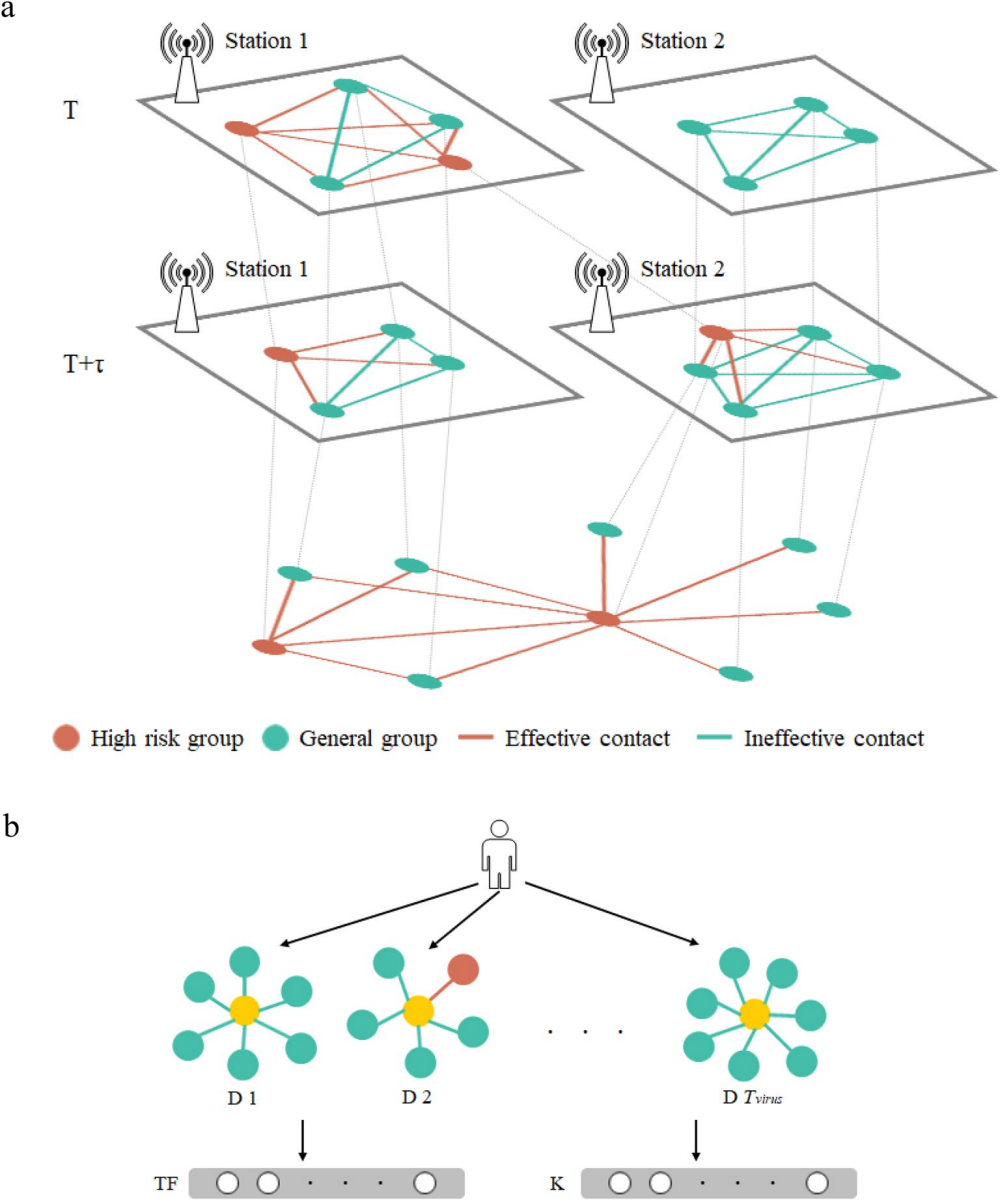
Contact networks structure. (**a**) Schematic diagram of sub-networks and contact network, taking two base stations as an example. Note that this figure only shows the trajectory simulation of two high-risk individuals and seven general individuals during two time slices, but in fact, each contact network is composed of $24/\tau$ sub-networks of all base stations. (**b**) Visualization of individual-centered contact feature sequence transformation. Before learning the transmission risk, the model takes each individual as an observation object and extracts the contact information of the adjacent nodes from the contact networks within $T_{virus}$.

In order to simulate the risks of spread infectious diseases in the crowd better, we proposed a growing network based on the microscopic spatiotemporal contact details among individuals, which called contact network. In this contact network, every mobile phone user is a node. Only when the effective contact occurs, the corresponding nodes will form an edge and the weight of the edge is their contact strength. As shown in Fig. 1a, the red dots indicate individuals of high-risk group and green dots indicate individuals of general group. At time *T*, there are two high-risk individuals under Station 1 and they have effective contacts (red line) with other people under the same station; while the other contacts are invalid (green line). And under Station 2, all people are belonging to the general group, so there is no effective contact. Thus, each base station forms a sub-network. After a time slice τ, some individuals move from one station to another, and then each base station generate a new sub-network following by the latest contacts. With people moving across the base stations during one day, such sub-network will be generated continuously. At the end of the day, all of the effective contacts and the nodes to which they are connected eventually form a daily contact network. Obviously, people who do not have contacted with the high-risk group are not included in the contact network.

Because the contact network describes the possible path of epidemic spreading in detail, we can further learn the transmission risk based on artificial neural network. The purpose of transmission risk learning is to identify individuals with higher potential infectious risk and estimate the corresponding probabilities. Here we mainly consider the first layer of virus transmission risks, that is, the infection between adjacent nodes in contact network. Therefore, as shown in Fig. 1b, all contact networks within nearly $T_{virus}$ days are transformed into individual-centered single-layer networks. $T_{virus}$ is the latent period of the infection and the potential risk of carrying virus can be taken into account by selecting the contact networks during the $T_{virus}$. And then, we extract contact feature sequences from those single-layer networks as the input of artificial neural network. Each contact feature sequence consists of two element sequences: $TF$, which represents the total contact strength, and $K$, which indicates whether the individual has contacted with the confirmed cases,

4 $$TF_{i,j,d} = \mathop \sum \limits_{{h \in H_{i,j,t} }} \omega_{h \to i,d}$$

5 $$K_{i,j,d} = \left\{ {\begin{array}{*{20}c} 1 & {if\ there\ are\ confirmed\ cases\ in\ H_{i,j,d} } \\ 0 & {otherwise} \\ \end{array} } \right.$$

where $i$ is an individual, $j$ is the municipal district of the city to be estimated and $d$ is the time. Thus, $TF_{i,j,d}$ indicates the contact intensity between individual $i$ and high-risk group in area $j$ on day $d$, which is the sum of edge weights of corresponding nodes in contact network. $H_{i,j,d}$ is the subset of high-risk group who had contact with individual $i$ in area $j$ on day $d$ effectively. If there is a confirmed case in subset $H_{i,j,d}$, then $K_{i,j,d}$ equals 1, otherwise it is 0.

### Artificial neural network of extreme events

Artificial neural network is used to learn epidemic transmission risk. After completing the feature transformation of contact network nodes, we calculate the cross term of contact intensity $TF$ and contact tag $K$. These three variables are standardized and then used as the input variables of the neural network. And then, we label the high-risk people by the potential risks' sources. Those isolated people are divided into two categories according to whether they had a sojourn to epidemic hot zone. If people have not been to the epidemic hot zone, their infection risks come from the contact in the observing area. In contrast, people who have been to the epidemic hot zone, the regional transmission risk comes from the epidemic hot zone people inflow. The rest individuals of the high-risk group are labeled as the third category.

**Figure 2:**
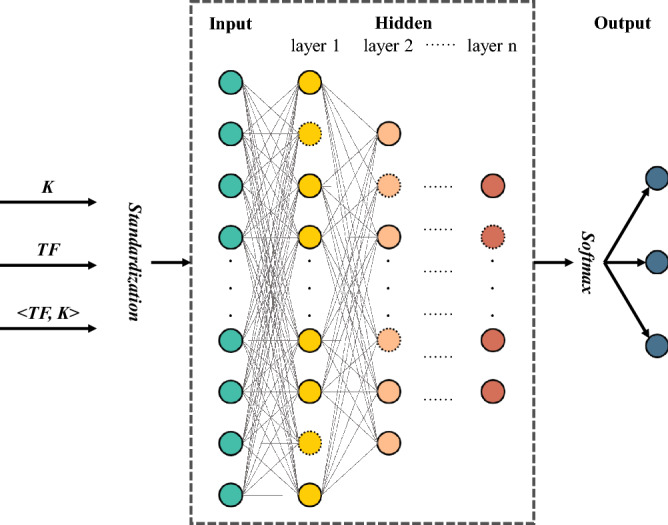
Artificial neural network structure. Visualization of neural network learning. After max–min scaling the input variables, the contact features can be learned by a fully-connected neural network. During model training, some neurons (dotted dots) are temporarily discarded from the network according to a certain probability, so that the network can avoid over fitting and be generalized better.

As shown in Fig. 2, the basic framework of the network is fully-connected and adopts leaky ReLU as activation function to reduce the silent neurons. However, isolation is an extreme event, that is, the proportion of positive-marked data in the dataset is very low. The high-risk group accounts for a very small number of the total population, let alone those who are isolated. Due to the imbalance of three kinds of people, it is necessary to adjust the neural network in the multi-classification training^61–63^. Therefore, in order to avoid the prediction error of the true positive cases caused by imbalanced data training, the neural network adopts a weighted cross entropy $L\left( {Y,P} \right)$ as the loss function for extreme event learning,

6 $$L\left( {Y,P} \right) = - \frac{1}{N}\mathop \sum \limits_{i} \left( {w_{k} \mathop \sum \limits_{k} y_{i,k} {\text{log}}p_{i,k} } \right)$$

where $N$ is the size of training sample, $k$ marks different classes. $y_{i,k}$ indicates whether the individual $i$ belongs to class $k$, if so, it is 1; otherwise, it is 0. $p_{i,k}$ is the probability that the model predicts individual $i$ belonging to class $k$ and $w_{k}$ is the weight of class $k$.

This loss function can give larger weight to the rarer categories, that is to say, the corresponding $w_{k}$ of the isolated groups are larger in order to increase the misclassified cost of these two rare categories, so that the neural network can learn useful information more effectively and achieve better prediction results.

After normalizing the initial learning results of neural network by *Softmax*, the probability that individual $i$ belongs to each class can be obtained. The class with the largest probability is the prediction class of individual $i$.

### Estimation of regional transmission risk

The main residence of each individual is determined by their most frequently located region for mobile phone signals during the night. Thus, we can divide those people into different group in terms of their residences. The risks of infectious disease transmission will come from the activities of people living there.

Since we have labeled the high-risk people as three categories and used multi-classification learning to fit how likely these people are to belong to the certain category, risk due to epidemic hot zones people inflow and risk due to close contacts are the average probability of corresponding-labeled individuals settled here,

7 $$TR_{s}^{{\left( {inflow} \right)}} = \frac{1}{{N_{s} }}\mathop \sum \limits_{i = 0}^{{N_{s} }} p_{i,s}^{{\left( {ehz} \right)}}$$

8 $$TR_{s}^{{\left( {contact} \right)}} = \frac{1}{{N_{s} }}\mathop \sum \limits_{i = 0}^{{N_{s} }} p_{i,s}^{{\left( {non} \right)}}$$

where $s$ is the region of risk to be assessed, $N_{s}$ is the number of individuals settled in $s$. $p_{i,s}^{{\left( {ehz} \right)}}$ is the probability of disease transmission from epidemic hot zones caused by individual $i$ and $TR_{s}^{{\left( {inflow} \right)}}$ represents the risk caused by the inflow people from epidemic hot zones. Similarly, $p_{i,s}^{{\left( {non} \right)}}$ is the probability of disease transmission caused by individual $i$ who have not been to the epidemic hot zones and $TR_{s}^{{\left( {contact} \right)}}$ represents the risk caused by the close contacts within the observing region. It is obvious that $TR_{s}^{{\left( {inflow} \right)}}$ and $TR_{s}^{{\left( {contact} \right)}}$ are between 0 and 1, and larger values mean higher regional transmission risks.

Because of the properties of the *Softmax* function, the probabilities of no risk and other two risks are additive, and the sum of them is equal to one. Thus, the total transmission risk can be derived from $TR_{s}^{{\left( {inflow} \right)}}$ and $TR_{s}^{{\left( {contact} \right)}}$,

9 $$TR_{s} = TR_{s}^{{\left( {inflow} \right)}} + TR_{s}^{{\left( {contact} \right)}} = \frac{1}{{N_{s} }}\mathop \sum \limits_{i = 0}^{{N_{s} }} \left( {p_{i,s}^{{\left( {ehz} \right)}} + p_{i,s}^{{\left( {non} \right)}} } \right)$$

$TR_{s}$ ranges likewise from zero to one. Because this is a bottom-up indicator, the regional transmission risk will rise if individuals are more likely to be classified into potential isolated group. In the contrast, if most individuals are predicted as the non-isolated group, the regional transmission risk will decrease.

### Data

We intercepted China Telecom's mobile signaling data in Shanghai from January 22 to February 4, 2020 to capture the users' real-time trajectories. We divided these 7,451,621 mobile phone users into high-risk group and general group according to their epidemiological diagnosis and historical action trails. High-risk group includes four kinds of people: the confirmed cases, the suspected cases, the medical isolators other than the first two and the people who once had a sojourn to epidemic hot zone. Considering the features of epidemic transmission and population flow in the early stage of COVID-19, forty-eight cities in China, including Wuhan and Wenzhou, were marked as the high-risk epidemic hot zones (see more details in Supplementary Information). And then, we identified 735,546 high-risk users in Shanghai based on mobile phone tracking during this period. In addition to the high-risk group, the rest of mobile phone users belonged to the general group.

As of February 4, 2020, there were 22,501 people in the isolation list provided by Shanghai Center for Disease Control and Prevention, covering eight districts in Shanghai. This eight districts include Baoshan, Chongming, Hongkou, Huangpu, Minhang, Pudong, Songjiang and Xuhui. Among them, 2459 isolators on the list were effectively matched, accounting for only 0.3343% of the Telecom high-risk users. In these matched isolators, 1742 isolators had epidemic hot zone sojourn and 717 isolators did not leave Shanghai during the observing period, accounting for 0.2368% and 0.0975% of the Telecom high-risk users respectively. For those users, being isolated was indeed a rare event.

## Results

### Crowd contact based on mobile phone tracking

**Figure 3:**
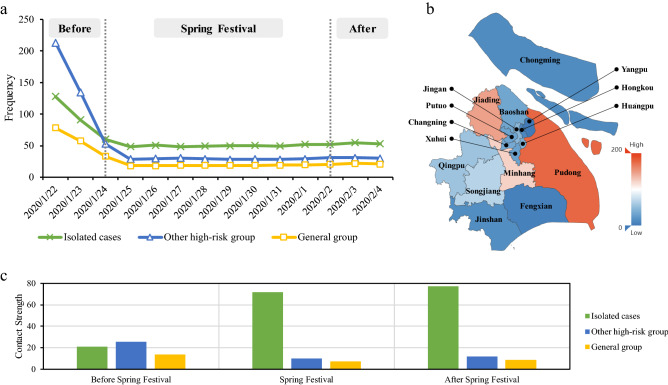
Crowd contact features of COVID-19 in the early stage. (**a**) The daily effective contact frequency per capita. The Spring Festival holiday in 2020 was originally from January 24 to January 30, then extended to February 2 due to COVID-19. On January 23, Wuhan announced the lockdown of the city and other local governments called on people to reduce unnecessary outdoor activities and maintain social distance. (**b**) Map of high-risk group’s average effective contact frequency. (**c**) Contact strength before and after the Spring Festival. We divided the fourteen days into three slots: before the Spring Festival (January 22–January 23), the Spring Festival (January 24–February 2) and after the Spring Festival (February 3–February 4).

According to the contact network, we can obtain the crowd contact characteristics on time trend, regional distribution and different groups. Generally, the spatiotemporal contact characteristics are consistent with the situation in Shanghai at that time, which also confirm the rationality of the contact network. In these 14 days, each high-risk individual was exposed to effective contact 51.81 times a day on average, while that of individuals in the general group was only 27.33 times. As shown in Fig. 3a, the effective contact frequencies of all isolators, non-isolated high-risk group and general group showed L-shaped as a whole. These curves declined at the beginning, and January 24 was a turning point. Since then, the curves have tended to be stable. On January 22, the effective contact frequency of each non-isolated high-risk individual was 213.36 times, which was higher than that of isolated group. However, the situation reversed since the Spring Festival. The daily effective contact frequency of non-isolated high-risk group has been lower than that of isolators, and closed to that of general group, maintained at about 30 times per capita. After February 3, people returned to work, and there was no apparent rebound in the effective contact frequency in Shanghai. This indicated that the policies of tighten travel restriction and keeping social distance called for by the government were well implemented. The average effective contact frequency of high-risk group (Fig. 3b) in Pudong was the highest, accounting for 176.36 times a day, which was partly due to the huge inter-cities population flow of Pudong Airport. Similarly, the effective contact frequency in Minhang, which has another airport and Hongqiao Railway Station with the largest passenger traffic volume in Shanghai, was also very high. As an important industrial area in Shanghai and a vital highway transportation hub connecting Jiangsu Province, Jiading had an effective daily contact frequency of 147.10 times a day. In contrast, the effective contact frequencies of high-risk group in suburbs such as Chongming and Fengxian, and urban centers such as Yangpu and Hongkou were much lower.

Figure 3c illustrates the differences in contact strength among isolators, non-isolated high-risk group and general group in three slots. Before the Spring Festival, the contact strength of the three groups was relatively close. However, the government had taken the quarantine measures, adopted the travel restriction policy and appealed for keeping social distance successively since January 23. These actions brought that the non-isolated high-risk group and the general group have reduced the contact strength by more than half during the Spring Festival. Although there was a slight increase after returning to work on February 3, the contact strength still remained low. Owing to gathering for medical observation, the isolated cases' mobile phone signaling would be received by the same base station frequently, which caused incessant effective contacts. In addition, most isolators have a larger virus carrying risk coefficient. Therefore, even if the overall population mobility in Shanghai declined, the contact strength of isolators still increased during the observation period continuously.

### Neural network classification

After constructing the Shanghai contact networks, we trained the neural network under different hyper-parameter settings. We took the neural network with general cross entropy loss function as the baseline and compared the classification results of the neural network with weighted cross entropy loss function with it (Table 1). Except for the loss function, the neural network structure of baseline is the same as that adopted in our extreme events model. Our classification goal is to accurately find true positives, but people who are really likely to be infected account for a small part of the population. A large proportion of negative cases will make many indicators such as accuracy fail. For example, even if all positive cases are classified as negative, accuracy will equal the proportion of negative cases in the samples and the result will show very well. In our data set, the accuracy will never be lower than 99%, which makes no sense to measure the quality of the model. Conversely, recall can evaluate whether all actual positive examples have been predicted and can support our study objectives better.

**Table 1:** Recall of COVID-19 exposure risk in test set.

	$l$	Weight*				Baseline
		20%	40%	60%	80%	
Isolator with epidemic hot zone sojourn	0.01	70.85%	64.57%	63.82%	65.33%	1.89%
	0.02	57.94%	57.67%	60.85%	57.41%	
	0.03	54.32%	54.59%	60.27%	65.14%	
Isolator without epidemic hot zone sojourn	0.01	18.35%	27.52%	24.77%	31.19%	0.00%
	0.02	16.36%	16.36%	18.18%	18.18%	
	0.03	18.70%	21.95%	20.33%	15.45%	

*in order to study the influence of the loss function’s weight on the exposure risk prediction better, the weights of rare categories are not set as a constant value, but set as the proportion of training set sample size.

The baseline recalls of two isolated group are only 1.89% and 0.00% respectively. However, by using the advanced extreme event neural network model, 70.85% of the isolators with a sojourn to epidemic hot zone can be identified successfully. Even if the highest recall of isolators without sojourn to epidemic hot zone is only 31.19%, it is still significantly higher than that of baseline. The results show that our improved model is superior to the general neural network in extreme event prediction and can effectively identify the individuals who are included in the Shanghai CDC isolation list due to the different ways of contacts. According to the ablation experiment results, the model with Leaky ReLU slope of 0.01 and rare category weight proportion of 20% was selected to predict the exposure risk of all individuals.

### COVID-19 transmission risks in shanghai

**Figure 4:**
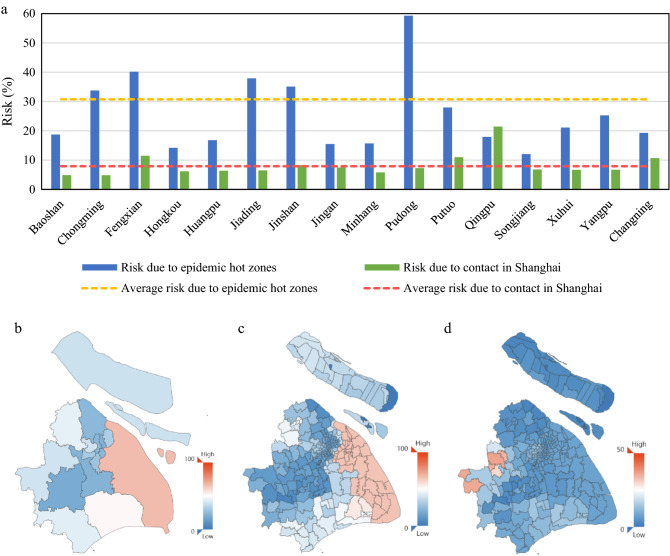
COVID-19 transmission risks in Shanghai. (**a**) Two kinds of COVID-19 transmission risks in 16 districts of Shanghai. (**b**) Map of Shanghai total transmission risk in district level. Baoshan, Jiading and Qingpu locate in the west of Shanghai, bordering Jiangsu Province; while Qingpu and Jinshan border on Zhejiang Province. According to Baidu Migration Index, Jiangsu and Zhejiang are the two major provinces of immigration to Shanghai from January 22 to February 4, 2020 (see more details in Supplementary Information). (**c**) Map of street-level transmission risk due to epidemic hot zones people inflow. (**d**) Map of street-level transmission risk due to close contacts within Shanghai.

Since we have labeled the isolators into two categories, the regional transmission risk can be divided into the following two kinds correspondingly: one is the risk caused by the inflow of people from epidemic hot zones, and the other is the risk caused by close contacts within Shanghai. Figure 4a shows two types of COVID-19 transmission risks in Shanghai. As a whole, Shanghai transmission risk due to the epidemic hot zones' people inflow was 30.76%, among which Pudong, Fengxian, Jiading, Jinshan and Chongming exceed the city average risk. In contrast, the transmission risk due to epidemic hot zones in Songjiang was the lowest, only 12.07%. That's because Songjiang has a college town and large-scale industrial areas, a large number of students and migrant workers returned home as early as before the Spring Festival, and did not return until the observing period. Besides, Hongkou and Jing'an, which are located in the center of the city, have relatively low transmission risk due to the inflow from epidemic hot zones, which was about 15%. Meanwhile, the COVID-19 transmission risk due to close contacts in Shanghai was 7.9%, among which Qingpu, Putuo, Fengxian, Changning and Jinshan exceed the city average level. It is worth noting that the Shanghai Public Health Clinical Center is located in Jinshan, where all isolators get the medical care. The centralized medical isolation may be one of the critical reasons for the high transmission risk caused by close contacts in Jinshan.

It can be seen from Fig. 4b that the areas with high total transmission risk of COVID-19 were mainly concentrated at the border of Shanghai. Pudong’s total risk was particularly high, reaching 66.55%. On the contrary, the total transmission risk in the center of Shanghai was relatively low. In terms of risk due to inflow from epidemic hot zones, the transmission risks in suburban streets were much higher than that in urban (Fig. 4c), especially the eastern, southern and northwestern borders of Shanghai. The streets of Pudong in particular deserve mention ─ the transmission risks from epidemic hot zones of most streets were all greater than 60% except for Lujiazui and other minority areas. The streets with high risk due to close contact (Fig. 4d) were mainly concentrated in the west of Shanghai, and Xianghuaqiao street of Qingpu had the highest risk, with a risk of 36.51%. In addition, some streets located in urban, such as Caoyangxincun street and Ganquanlu street, also have high risk, reaching 13.51% and 13.40% respectively.

## Discussion and conclusion

In this paper, a regional epidemic transmission risk precaution based on machine learning is proposed. Firstly, we distinguish whether individuals appear at the same time through the trajectories recorded by their mobile phones and construct the contact networks according to the way they contact. Then, the contact network is transformed into an individual-centered contact feature matrix, and the extreme event neural network is used to classify the isolated people. Finally, according to the classification results, we select the optimal model to predict the probability of each individual becoming a high-risk infected person and estimate the regional transmission risks.

We conducted a large-scale experiment with about 7.5 million people in Shanghai at the beginning of the COVID-19 outbreak in 2020. In the case of extremely imbalanced samples, the model can predict the rare categories effectively, and the recall can reach more than 70% among the isolators with epidemic hot zone sojourn. However, the recall of the isolators without high-risk areas sojourn history is only 31.19%, but it is still higher than that predicted by general neural network. On the one hand, this kind of isolators only accounts for 0.0975% of the samples. The scarcity of such isolators not only makes it difficult to capture their contact features, but also the proportions of various groups in the data set will be seriously unbalanced, which also can easily lead to model misjudgment. On the other hand, the coverage of the sample is insufficient. Considering that there were 40.92 million mobile phone users in Shanghai in 2020, the sample of China Telecom's mobile phone users is even less than one-fifth of Shanghai's mobile phone market. Nevertheless, the COVID-19 cases used in this study only cover half of Shanghai and the cases in the other eight districts are not taken into account. Due to the limitation of experimental data, the whole population's contact situation in Shanghai was not fully described when constructing contact network, which will have a negative impact on the prediction results of the model. However, as a whole, the precaution framework is of great significance for the regional transmission risk estimation of COVID-19 and other similar epidemics.

Artificial intelligence has been widely adopted in many fields in our real life^64–66^, including the prevention and control of infectious diseases. Different from the previous studies on epidemic transmission through wearable devices or mobile phones, this machine learning based regional epidemic transmission risk precaution is completely bottom-up and can be used for early warning of regional epidemic on the premise of anonymity. When facing the changes of regional isolation and flow restriction policies^67,68^, which are very common in reality, this method has better flexibility and can make self-adaptive adjustment. In addition, using mobile phone signaling to estimate the risk of regional epidemic spread can provide effective auxiliary information support for government policy making and epidemic prevention work with high efficiency and low cost. Especially for low-income and middle-income countries, it can alleviate the financial difficulties caused by epidemic prevention and control. In order to implement effective intervention measures, it requires close interaction between policy makers and model prediction during the outbreak of epidemic^69^. But, remarkably, digital governance has raised the global concern on the citizens' privacy protection when using public data^70–72^. Therefore, all countries need to strictly abide by the data privacy law when using trajectory data and the scope of data usage should be limited in accordance with the minimization principle, including obtaining the explicit consent of users, collecting as little information as possible and ensuring data security. At the same time, the data and information holders should guarantee the data privacy through emerging technology, such as desensitizing data, and reduce the possibility of data abuse from the source^73^.

As mentioned above, this method was proposed for regional epidemic transmission risk precaution. Its main purpose is to provide early warning before a large-scale epidemic outbreak and provide auxiliary information to decision makers. Labor loss, production suspension, trade obstruction, and rising market uncertainty may all become the consequences of national epidemic prevention policies. If the governors cannot balance the control measures and economic pressures well, an economic crisis may follow the pandemic^74^. By controlling the risk before the virus spreads widely, governors can moderate the enormous economic and social disruption caused by control measures for infectious diseases. Thus, the research design mainly focuses on the contact network and extreme events classification. On the one hand, we pay attention to the inflow risk from the external epic hot zone when calculating the contact strength; on the other hand, the transmission risk has a relatively long window period (14 days in the experiment), which has an impact on the contact strength and the individual centered contact feature. As a proactive prevention and control method, the best time for it to work is when there are only a few infected people because of the aforementioned mechanism design. Generally, the limitation of this risk precaution is that when a large-scale and mass outbreak occurs in the city, such as the Omicron virus pandemic in Shanghai in the spring of 2022, its early warning effect will be greatly reduced. The outbreak of the Omicron virus pandemic this time is so sudden that social resources such as the CDC, public health departments, communication operators and so on are fully occupied. Therefore, it is worthy to retrospectively analyze the differences of these two outbreaks in the future.

## Supplementary Information

Supplementary Information.

## Data Availability

The data that support the findings of this study are available from Shanghai Ideal Information Industry (Group) Co., LTD but restrictions apply to the availability of these data, which were used under license for the current study, and so are not publicly available. Data are however available from the authors upon reasonable request and with permission of Shanghai Ideal Information Industry (Group) Co., LTD.
