# Supplementary material for: Machine learning based regional epidemic transmission risks precaution in digital society
Source: Sci Rep. 2022 Nov 28;12:20499. doi: 10.1038/s41598-022-24670-z (PMC9705289; doi:10.1038/s41598-022-24670-z)
Supplement: Supplementary file 1 — Supplementary Information. [file 41598_2022_24670_MOESM1_ESM.docx]

Supplementary Information

Machine Learning Based Regional Epidemic Transmission Risks Precaution in Digital Society

Zhengyu Shi^a^, Haoqi Qian^b,c,d,^^[[1]](#footnote-1)^, Yao Li^e^, Fan Wu^f,g^, Libo Wu^h,i,d,^^[[2]](#footnote-2)^,

^a^School of Data Science, Fudan University, Shanghai 200433, China

^b^Institute for Global Public Policy, Fudan University, Shanghai 200433, China

^c^LSE-Fudan Research Centre for Global Public Policy, Fudan University, Shanghai 200433, China

^d^ MOE Laboratory for National Development and Intelligent Governance, Fudan University, Shanghai 200433, China

^e^Shanghai Ideal Information Industry (Group) Co., Ltd, Fudan University, Shanghai 200120, China

^f^Shanghai Public Health Clinical Center, Fudan University, Shanghai 200032, China

^g^Key Laboratory of Medical Molecular Virology, Fudan University, Shanghai 200032, China

^h^School of Economics, Fudan University, Shanghai 200433, China

^i^Institute for Big Data, Fudan University, Shanghai 200433, China

S1. High-Risk Epidemic Hot Zones

Considering the COVID-19 infection situation in China from January 22 to February 4, 2020, we marked 48 cities as high-risk epidemic hot zones. Table S1 shows those cities and the risk coefficients correspondingly.

Table S1 High-risk epidemic hot zones and risk coefficients

|  | Province | City | Cumulative number of confirmed cases | Permanent resident population (unit: 10,000) | Risk coefficient |
| --- | --- | --- | --- | --- | --- |
| 1 | Hubei | Wuhan | 18454 | 1108.1 | 133.6588795 |
| 2 | Hubei | Ezhou | 790 | 107.77 | 58.83225068 |
| 3 | Hubei | Xiaogan | 2642 | 492 | 43.09768723 |
| 4 | Hubei | Suizhou | 1095 | 221.67 | 39.6454592 |
| 5 | Hubei | Xiantao | 438 | 114 | 30.83582084 |
| 6 | Hubei | Huanggang | 2332 | 633 | 29.56726071 |
| 7 | Hubei | Huangshi | 835 | 247.07 | 27.12393624 |
| 8 | Hubei | Jingmen | 656 | 289.65 | 18.17676369 |
| 9 | Xinjiang | Urumqi | 20 | 9.41 | 17.05794174 |
| 10 | Hubei | Tianmen | 261 | 127.23 | 16.46407117 |
| 11 | Hubei | Xianning | 515 | 254.33 | 16.25159131 |
| 12 | Hubei | Jingzhou | 1075 | 559.02 | 15.43360472 |
| 13 | Hubei | Xiangyang | 1063 | 566.9 | 15.04918781 |
| 14 | Hubei | Yichang | 772 | 413.585 | 14.98093003 |
| 15 | Xinjiang | Turpan | 1 | 0.54 | 14.86252146 |
| 16 | Hubei | Shiyan | 505 | 340.6 | 11.89961715 |
| 17 | Hubei | Shennongjia | 10 | 7.67 | 10.46383519 |
| 18 | Hubei | Qianjiang | 90 | 96.6 | 7.47741763 |
| 19 | Jiangxi | Xinyu | 110 | 118.07 | 7.477206529 |
| 20 | Hunan | Changsha | 219 | 264.21 | 6.65244233 |
| 21 | Hunan | Yueyang | 134 | 175.67 | 6.122001782 |
| 22 | Hainan | Sanya | 45 | 77.39 | 4.666743397 |
| 23 | Hubei | Enshi | 195 | 337.8 | 4.632988484 |
| 24 | Hunan | Zhuzhou | 68 | 119.29 | 4.575000319 |
| 25 | Hunan | Loudi | 69 | 124.51 | 4.447655206 |
| 26 | Xinjiang | Bazhou | 3 | 5.8 | 4.151255995 |
| 27 | Zhejiang | Wenzhou | 474 | 921.5 | 4.128281056 |
| 28 | Xinjiang | Dijiushi | 4 | 8.2 | 3.915005653 |
| 29 | Guangdong | Zhuhai | 86 | 189.11 | 3.649809617 |
| 30 | Xinjiang | Yili | 10 | 22.5 | 3.567005151 |
| 31 | Hunan | Shaoyang | 91 | 216.82 | 3.368436051 |
| 32 | Hunan | Yiyang | 54 | 131.14 | 3.30479736 |
| 33 | Anhui | Bengbu | 134 | 339.2 | 3.170554401 |
| 34 | Hunan | Xiangtan | 32 | 83.89 | 3.061442018 |
| 35 | Hunan | Changde | 73 | 193.67 | 3.025148944 |
| 36 | Jiangxi | Nanchang | 195 | 546.35 | 2.864507202 |
| 37 | Henan | Xinyang | 228 | 647.4 | 2.826496204 |
| 38 | Ningxia | Wuzhong | 15 | 42.9154 | 2.80520335 |
| 39 | Xinjiang | Shihezi | 2 | 5.8 | 2.767503996 |
| 40 | Guangdong | Shenzhen | 375 | 1302.66 | 2.31039611 |
| 41 | Heilongjiang | Shuangyashan | 38 | 140.9 | 2.164506319 |
| 42 | Hunan | Yongzhou | 42 | 160.54 | 2.099676011 |
| 43 | Hainan | Changjiang | 6 | 23.35 | 2.062294199 |
| 44 | Zhejiang | Taizhou | 143 | 611.8 | 1.875913546 |
| 45 | Sichuan | Ganzi | 27 | 118.63 | 1.826650619 |
| 46 | Hunan | Huaihua | 39 | 172.8 | 1.811369803 |
| 47 | Hainan | Wanning | 13 | 57.86 | 1.803230222 |
| 48 | Heilongjiang | Jixi | 38 | 172.7 | 1.765946383 |

S2. Baidu Migration Index of Shanghai

Table S2 shows the Baidu Migration Index^[[3]](#footnote-3)^ top 10 cities of Shanghai from January 22 to February 4, 2020. In this period, the main inflow and outflow of people in Shanghai came from Jiangsu and Zhejiang Province.

Table S2 Baidu Migration Index of Shanghai

| Date | To Shanghai | | | From Shanghai | | |
| --- | --- | --- | --- | --- | --- | --- |
|  | City | Province | Ratio  (%) | City | Province | Ratio  (%) |
| Jan 22, 2020 | Suzhou | **Jiangsu** | 18.76 | Suzhou | **Jiangsu** | 4.33 |
|  | Hangzhou | **Zhejiang** | 5.04 | Nantong | **Jiangsu** | 4.28 |
|  | Jiaxing | **Zhejiang** | 4.69 | Yancheng | **Jiangsu** | 3.60 |
|  | Nantong | **Jiangsu** | 4.59 | Hefei | Anhui | 2.64 |
|  | Beijing | Beijing | 3.36 | Fuyang | Anhui | 2.56 |
|  | Wuxi | **Jiangsu** | 3.26 | Lu'an | Anhui | 2.20 |
|  | Ningbo | **Zhejiang** | 3.07 | Chuzhou | Anhui | 2.19 |
|  | Nanjing | **Jiangsu** | 2.77 | Huainan | Anhui | 2.07 |
|  | Changzhou | **Jiangsu** | 1.86 | Wuhu | Anhui | 2.01 |
|  | Yancheng | **Jiangsu** | 1.69 | Taizhou | **Jiangsu** | 1.87 |
| Jan 23, 2020 | Suzhou | **Jiangsu** | 16.65 | Nantong | **Jiangsu** | 5.10 |
|  | Hangzhou | **Zhejiang** | 4.86 | Suzhou | **Jiangsu** | 4.72 |
|  | Nantong | **Jiangsu** | 4.22 | Yancheng | **Jiangsu** | 3.84 |
|  | Jiaxing | **Zhejiang** | 4.15 | Fuyang | Anhui | 2.75 |
|  | Beijing | Beijing | 3.48 | Hefei | Anhui | 2.42 |
|  | Wuxi | **Jiangsu** | 3.11 | Wuhu | Anhui | 2.16 |
|  | Nanjing | **Jiangsu** | 2.84 | Lu'an | Anhui | 2.12 |
|  | Ningbo | **Zhejiang** | 2.64 | Huainan | Anhui | 2.03 |
|  | Changzhou | **Jiangsu** | 1.80 | Taizhou | **Jiangsu** | 1.99 |
|  | Hefei | Anhui | 1.63 | Yangzhou | **Jiangsu** | 1.70 |
| Jan 24, 2020 | Suzhou | **Jiangsu** | 16.91 | Suzhou | **Jiangsu** | 6.70 |
|  | Hangzhou | **Zhejiang** | 4.60 | Nantong | **Jiangsu** | 5.69 |
|  | Jiaxing | **Zhejiang** | 4.33 | Yancheng | **Jiangsu** | 4.01 |
|  | Nantong | **Jiangsu** | 3.99 | Fuyang | Anhui | 2.40 |
|  | Beijing | Beijing | 3.43 | Wuxi | **Jiangsu** | 2.26 |
|  | Wuxi | **Jiangsu** | 3.22 | Taizhou | **Jiangsu** | 2.22 |
|  | Nanjing | **Jiangsu** | 2.97 | Wuhu | Anhui | 2.09 |
|  | Ningbo | **Zhejiang** | 2.49 | Hangzhou | **Zhejiang** | 2.07 |
|  | Changzhou | **Jiangsu** | 1.69 | Jiaxing | **Zhejiang** | 1.97 |
|  | Guangzhou | Guangdong | 1.62 | Hefei | Anhui | 1.91 |
| Jan 25, 2020 | Suzhou | **Jiangsu** | 17.53 | Suzhou | **Jiangsu** | 8.39 |
|  | Nantong | **Jiangsu** | 5.89 | Nantong | **Jiangsu** | 5.61 |
|  | Jiaxing | **Zhejiang** | 5.07 | Yancheng | **Jiangsu** | 3.69 |
|  | Hangzhou | **Zhejiang** | 4.49 | Wuxi | **Jiangsu** | 2.51 |
|  | Wuxi | **Jiangsu** | 3.62 | Jiaxing | **Zhejiang** | 2.42 |
|  | Ningbo | **Zhejiang** | 2.71 | Hangzhou | **Zhejiang** | 2.39 |
|  | Nanjing | **Jiangsu** | 2.45 | Ningbo | **Zhejiang** | 2.16 |
|  | Beijing | Beijing | 2.24 | Fuyang | Anhui | 2.14 |
|  | Changzhou | **Jiangsu** | 2.07 | Taizhou | **Jiangsu** | 2.02 |
|  | Huzhou | **Zhejiang** | 1.65 | Wuhu | Anhui | 1.87 |
| Jan 26, 2020 | Suzhou | **Jiangsu** | 13.72 | Suzhou | **Jiangsu** | 9.65 |
|  | Nantong | **Jiangsu** | 6.27 | Nantong | **Jiangsu** | 6.00 |
|  | Huzhou | **Zhejiang** | 4.71 | Yancheng | **Jiangsu** | 4.16 |
|  | Hangzhou | **Zhejiang** | 4.46 | Jiaxing | **Zhejiang** | 2.74 |
|  | Jiaxing | **Zhejiang** | 4.03 | Wuxi | **Jiangsu** | 2.38 |
|  | Wuxi | **Jiangsu** | 3.92 | Hangzhou | **Zhejiang** | 2.24 |
|  | Ningbo | **Zhejiang** | 3.40 | Taizhou | **Jiangsu** | 2.14 |
|  | Yancheng | **Jiangsu** | 2.34 | Fuyang | Anhui | 1.99 |
|  | Changzhou | **Jiangsu** | 2.17 | Ningbo | **Zhejiang** | 1.92 |
|  | Shaoxing | **Zhejiang** | 1.86 | Yangzhou | **Jiangsu** | 1.77 |
| Jan 27, 2020 | Suzhou | **Jiangsu** | 8.52 | Suzhou | **Jiangsu** | 10.55 |
|  | Nantong | **Jiangsu** | 6.98 | Nantong | **Jiangsu** | 5.53 |
|  | Yancheng | **Jiangsu** | 3.73 | Yancheng | **Jiangsu** | 3.41 |
|  | Wuxi | **Jiangsu** | 2.80 | Jiaxing | **Zhejiang** | 2.71 |
|  | Hangzhou | **Zhejiang** | 2.75 | Wuxi | **Jiangsu** | 2.35 |
|  | Jiaxing | **Zhejiang** | 2.47 | Hangzhou | **Zhejiang** | 2.09 |
|  | Ningbo | **Zhejiang** | 2.40 | Fuyang | Anhui | 2.00 |
|  | Taizhou | **Jiangsu** | 2.25 | Ningbo | **Zhejiang** | 1.81 |
|  | Fuyang | Anhui | 1.86 | Taizhou | **Jiangsu** | 1.69 |
|  | Hefei | Anhui | 1.76 | Nanjing | **Jiangsu** | 1.65 |
| Jan 28, 2020 | Nantong | **Jiangsu** | 6.30 | Suzhou | **Jiangsu** | 11.82 |
|  | Suzhou | **Jiangsu** | 5.81 | Nantong | **Jiangsu** | 4.80 |
|  | Yancheng | **Jiangsu** | 4.74 | Jiaxing | **Zhejiang** | 3.14 |
|  | Fuyang | Anhui | 2.76 | Yancheng | **Jiangsu** | 2.29 |
|  | Taizhou | **Jiangsu** | 2.39 | Hangzhou | **Zhejiang** | 2.23 |
|  | Huainan | Anhui | 2.22 | Wuxi | **Jiangsu** | 2.16 |
|  | Lu'an | Anhui | 2.17 | Nanjing | **Jiangsu** | 1.89 |
|  | Hefei | Anhui | 2.14 | Ningbo | **Zhejiang** | 1.87 |
|  | Wuhu | Anhui | 1.83 | Beijing | Beijing | 1.68 |
|  | Yangzhou | **Jiangsu** | 1.78 | Fuyang | Anhui | 1.66 |
| Jan 29, 2020 | Nantong | **Jiangsu** | 6.26 | Suzhou | **Jiangsu** | 12.53 |
|  | Suzhou | **Jiangsu** | 5.59 | Nantong | **Jiangsu** | 4.11 |
|  | Yancheng | **Jiangsu** | 4.58 | Jiaxing | **Zhejiang** | 3.74 |
|  | Fuyang | Anhui | 2.94 | Hangzhou | **Zhejiang** | 2.26 |
|  | Huainan | Anhui | 2.35 | Wuxi | **Jiangsu** | 2.14 |
|  | Lu'an | Anhui | 2.34 | Yancheng | **Jiangsu** | 2.01 |
|  | Taizhou | **Jiangsu** | 2.24 | Ningbo | **Zhejiang** | 2.01 |
|  | Hefei | Anhui | 2.22 | Nanjing | **Jiangsu** | 2.00 |
|  | Bengbu | Anhui | 1.73 | Beijing | Beijing | 1.73 |
|  | Wuxi | **Jiangsu** | 1.70 | Hefei | Anhui | 1.53 |
| Jan 30, 2020 | Nantong | **Jiangsu** | 5.48 | Suzhou | **Jiangsu** | 13.89 |
|  | Suzhou | **Jiangsu** | 5.05 | Nantong | **Jiangsu** | 3.94 |
|  | Yancheng | **Jiangsu** | 4.36 | Jiaxing | **Zhejiang** | 3.88 |
|  | Fuyang | Anhui | 3.28 | Hangzhou | **Zhejiang** | 2.44 |
|  | Huainan | Anhui | 2.77 | Ningbo | **Zhejiang** | 2.08 |
|  | Lu'an | Anhui | 2.62 | Nanjing | **Jiangsu** | 2.01 |
|  | Hefei | Anhui | 2.14 | Wuxi | **Jiangsu** | 1.96 |
|  | Taizhou | **Jiangsu** | 2.05 | Yancheng | **Jiangsu** | 1.95 |
|  | Bozhou | Anhui | 2.03 | Beijing | Beijing | 1.88 |
|  | Wuhu | Anhui | 1.86 | Zhoushan | **Zhejiang** | 1.81 |
| Jan 31, 2020 | Suzhou | **Jiangsu** | 5.00 | Suzhou | **Jiangsu** | 15.19 |
|  | Nantong | **Jiangsu** | 4.58 | Nantong | **Jiangsu** | 4.23 |
|  | Fuyang | Anhui | 3.80 | Jiaxing | **Zhejiang** | 4.22 |
|  | Yancheng | **Jiangsu** | 3.73 | Hangzhou | **Zhejiang** | 2.63 |
|  | Huainan | Anhui | 2.76 | Ningbo | **Zhejiang** | 2.20 |
|  | Lu'an | Anhui | 2.57 | Wuxi | **Jiangsu** | 2.11 |
|  | Bozhou | Anhui | 2.09 | Yancheng | **Jiangsu** | 2.06 |
|  | Hefei | Anhui | 2.06 | Zhoushan | **Zhejiang** | 2.01 |
|  | Wuhu | Anhui | 1.87 | Beijing | Beijing | 1.94 |
|  | Taizhou | **Jiangsu** | 1.78 | Nanjing | **Jiangsu** | 1.93 |
| Feb 1, 2020 | Suzhou | **Jiangsu** | 5.33 | Suzhou | **Jiangsu** | 15.53 |
|  | Nantong | **Jiangsu** | 5.32 | Nantong | **Jiangsu** | 4.99 |
|  | Yancheng | **Jiangsu** | 4.21 | Jiaxing | **Zhejiang** | 4.77 |
|  | Fuyang | Anhui | 3.15 | Hangzhou | **Zhejiang** | 2.89 |
|  | Huainan | Anhui | 2.54 | Zhoushan | **Zhejiang** | 2.35 |
|  | Lu'an | Anhui | 2.49 | Wuxi | **Jiangsu** | 2.13 |
|  | Taizhou | **Jiangsu** | 2.24 | Ningbo | **Zhejiang** | 2.04 |
|  | Hefei | Anhui | 2.08 | Yancheng | **Jiangsu** | 1.98 |
|  | Wuhu | Anhui | 1.91 | Nanjing | **Jiangsu** | 1.90 |
|  | Yangzhou | **Jiangsu** | 1.78 | Beijing | Beijing | 1.80 |
| Feb 2, 2020 | Nantong | **Jiangsu** | 5.53 | Suzhou | **Jiangsu** | 16.27 |
|  | Suzhou | **Jiangsu** | 5.02 | Nantong | **Jiangsu** | 5.40 |
|  | Yancheng | **Jiangsu** | 3.74 | Jiaxing | **Zhejiang** | 4.99 |
|  | Fuyang | Anhui | 3.09 | Hangzhou | **Zhejiang** | 2.95 |
|  | Lu'an | Anhui | 2.37 | Yancheng | **Jiangsu** | 2.63 |
|  | Huainan | Anhui | 2.24 | Wuxi | **Jiangsu** | 2.34 |
|  | Taizhou | **Jiangsu** | 2.15 | Zhoushan | **Zhejiang** | 2.24 |
|  | Hefei | Anhui | 2.11 | Ningbo | **Zhejiang** | 2.00 |
|  | Wuhu | Anhui | 1.96 | Nanjing | **Jiangsu** | 1.98 |
|  | Anqing | Anhui | 1.86 | Beijing | Beijing | 1.82 |
| Feb 3, 2020 | Suzhou | **Jiangsu** | 5.75 | Suzhou | **Jiangsu** | 16.09 |
|  | Nantong | **Jiangsu** | 5.06 | Nantong | **Jiangsu** | 5.88 |
|  | Yancheng | **Jiangsu** | 3.92 | Jiaxing | **Zhejiang** | 4.60 |
|  | Fuyang | Anhui | 3.21 | Hangzhou | **Zhejiang** | 2.94 |
|  | Huainan | Anhui | 2.35 | Zhoushan | **Zhejiang** | 2.76 |
|  | Lu'an | Anhui | 2.12 | Yancheng | **Jiangsu** | 2.74 |
|  | Taizhou | **Jiangsu** | 1.96 | Nanjing | **Jiangsu** | 2.40 |
|  | Hefei | Anhui | 1.95 | Wuxi | **Jiangsu** | 2.35 |
|  | Wuhu | Anhui | 1.76 | Beijing | Beijing | 2.33 |
|  | Xuzhou | **Jiangsu** | 1.75 | Ningbo | **Zhejiang** | 2.06 |
| Feb 4, 2020 | Suzhou | **Jiangsu** | 5.79 | Suzhou | **Jiangsu** | 19.67 |
|  | Nantong | **Jiangsu** | 5.63 | Nantong | **Jiangsu** | 5.74 |
|  | Yancheng | **Jiangsu** | 3.79 | Jiaxing | **Zhejiang** | 5.08 |
|  | Fuyang | Anhui | 3.15 | Zhoushan | **Zhejiang** | 2.92 |
|  | Huainan | Anhui | 2.36 | Yancheng | **Jiangsu** | 2.84 |
|  | Hefei | Anhui | 2.02 | Hangzhou | **Zhejiang** | 2.61 |
|  | Lu'an | Anhui | 2.01 | Wuxi | **Jiangsu** | 2.36 |
|  | Wuhu | Anhui | 1.95 | Ningbo | **Zhejiang** | 2.04 |
|  | Taizhou | **Jiangsu** | 1.91 | Nanjing | **Jiangsu** | 2.03 |
|  | Anqing | Anhui | 1.72 | Hefei | Anhui | 1.65 |

1. Corresponding author, Email: qianhaoqi@fudan.edu.cn. [↑](#footnote-ref-1)
2. Corresponding author, Email: wulibo@fudan.edu.cn.

   We declare that we have no conflict of interest. [↑](#footnote-ref-2)
3. Baidu Migration Index: https://qianxi.baidu.com [↑](#footnote-ref-3)
